# Experimental and Numerical Study on the Strength of Aluminum Extrusion Welding

**DOI:** 10.3390/ma8074389

**Published:** 2015-07-17

**Authors:** Sedat Bingöl, Atilla Bozacı

**Affiliations:** 1 Department of Mechanical Engineering, Dicle University, 21280 Sur, Diyarbakir, Turkey; 2 Department of Mechanical Engineering, Yıldız Technical University, 34349 Beşiktaş, Istanbul, Turkey; E-Mail: abozaci@yildiz.edu.tr

**Keywords:** extrusion, seam weld, aluminum, ram speed, FEM, simulation

## Abstract

The quality of extrusion welding in the extruded hollow shapes is influenced significantly by the pressure and effective stress under which the material is being joined inside the welding chamber. However, extrusion welding was not accounted for in the past by the developers of finite element software packages. In this study, the strength of hollow extrusion profile with seam weld produced at different ram speeds was investigated experimentally and numerically. The experiments were performed on an extruded hollow aluminum profile which was suitable to obtain the tensile tests specimens from its seam weld’s region at both parallel to extrusion direction and perpendicular to extrusion direction. A new numerical modeling approach, which was recently proposed in literature, was used for numerical analyses of the study. The simulation results performed at different ram speeds were compared with the experimental results, and a good agreement was obtained.

## 1. Introduction

The extrusion process is a viable metal forming process that can provide the desired shapes of products that have high strength-to-weight ratios and good dimensional accuracy [1,2,3]. Extrusion welding takes place in hollow profiles made of lightweight alloys for many industrial applications using a porthole die or a die with a mandrel. In the hot extrusion process, the billet material is divided into metal streams by a porthole die, and these metal streams flow around the core supports that hold the stationary mandrel, and they are joined under pressure within the welding chamber [4]. Complex hollow or semi-hollow shapes can be produced in this way. Extrusion welding takes place when the required pressure value for a given temperature is applied within the welding chamber.

Some studies reported in literature were focused on the quality of extrusion welding. Chen *et al.* [5] performed analysis and designed porthole dies for a hollow complex extrusion. They proposed a design route of a multi-hole porthole die for hollow and thin-walled profiles. Zhao *et al.* [6] conducted multi-objective optimization of a complex hollow profile for porthole die extrusion based on a modern intelligent algorithm. They proposed a design procedure for practical extrusion considering several variables. Li *et al.* [7] analyzed the effect of the extrusion temperature on the integrity of a weld, and they showed that temperature was not a critical parameter with respect to the quality of a weld. According to Donati and Tomesani [8], the methods that are used most often to test the quality of extrusion welding are bending and tearing the seam welded profiles. These methods can be used only for dies that already have been manufactured, which is a typical trial-and-error approach. Therefore, there is still a need for a more systematic approach for predicting the welding quality.

Finite Element Methods (FEM), in parallel with physical modeling and analyses, assist in the design and optimization of extrusion processes. These numerical modeling methods allow localized process state variables to be calculated properly step by step for any given moment of the process [9,10]. However, actual material bonding, such as in extrusion welding, was not accounted for in the past by the developers of finite element (FE) software packages. Ceretti and Giardini [11] discussed this deficiency and suggested an algorithm to use with DEFORM™ 2D. However, the method they developed cannot generate localized state variables. Xu and Misiolek [12] proposed a new modeling approach to overcome this limitation. They virtually inserted a very thin fixed plate in the interface of the seam welds by attaching it to the short mandrel of the porthole die. The fixed pressure plate functioned as a sensor to record the information at the welding line. The results they reported from their study, which were obtained from a fixed plate, had high noise because they used a fixed step number that represented only a momentary state of the ram’s travel. Alharthi *et al.* [13] modified the Xu and Misiolek’ model by changing step number during the ram’s travel in the simulation to prevent the high noise results. However, the model’s performance used in the works of Xu and Misiolek [13] and Altharthi *et al.* [12] was not verified with mechanical tests. Therefore, in the current study, firstly, a seam welded extrusion profile which was suitable to obtain the test specimens from it as parallel and perpendicular to extrusion direction was produced at different ram speeds in a real extrusion company. Then, the specimens were machined and subjected to tensile test. Finally, the FEM simulations by using the fixed pressure plate were performed at the same speeds as in the extrusion process. The normal pressure and effective stress obtained from the fixed plate in the simulations were used to analyze the *P*/σ ratio, which is responsible for the welding quality in porthole extrusion. The welding line which is formed during the extrusion of the profiles was modeled using plane-strain criteria to examine whether proposed FE model displays the same trend as in experiments.

## 2. Materials and Method

### 2.1. Experimental Study

**Figure 1:**
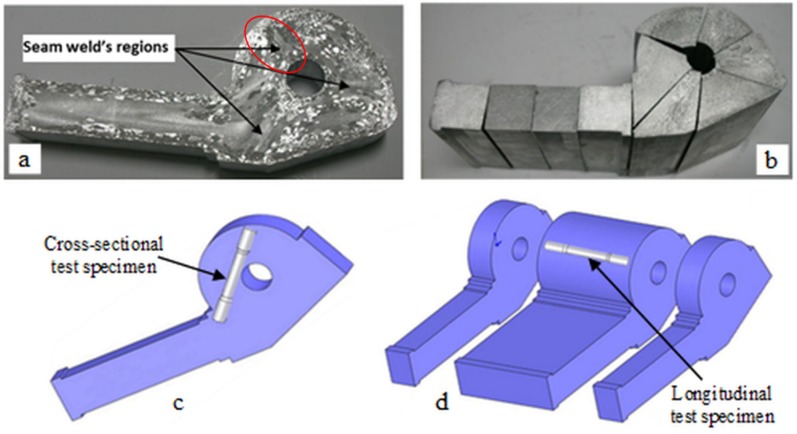
Preparing the seam welded tensile test specimens: (**a**) Macro etched specimen to detect seam welds; (**b**) Cutting the seam welded tensile specimens parallel to extrusion direction; (**c**) Illustration of the seam welded tensile specimen perpendicular to extrusion direction; (**d**) Illustration of the specimen parallel to extrusion direction.

The experimental hollow extrusion profile was produced by direct extrusion method in Sistem Aluminum Company (Istanbul, Turkey). The sectional area of the billet was 31,684 mm^2^, while that of the outlet was 6326 mm^2^, and the extrusion ratio was approximately 5. AA6063 alloy (consisted of 0.4 Si, 0.19 Fe, 0.008 Cu, 0.004 Mn, 0.48 Mg, 0.01 Zn, 0.005 Ti, and 0.002 Cr in wt % values) was used as billet material. The extruded profile was produced at different ram speeds of 2, 4, 6, 10 and 15 mm/s. Macro etching process was performed to see the seam weld regions on the produced profile (Figure 1a), Macro etching process were ground with 120, 180, 240, 400 and 600 grit abrasives successively, then the entire surfaces of them were etched by Poulton etching which provided the best result among some tried etching methods. The etching contains 60% HCI, 30% HNO_3_, 5% HF and 5% distilled water. After macro etching of the profile, the tensile test specimens with seam weld were prepared based on both parallel to extrusion direction and perpendicular to extrusion direction (Figure 1). The specimens were taken from profiles as extruded. Tensile tests were repeated at least three times and their average was taken into account.

### 2.2. Material Modeling in Hot Extrusion of AA6063

For numerical simulation of the metal forming process for high temperatures and strain rates, the acceptable extrapolations based on trusted constitutive models must be used. In this work, the Sellars-Tegart model, one of the most extensively used models, was considered appropriate to describe the behavior of AA6063:

(1) $$\text{σ}=\frac{1}{\text{β}}\;{\text{sinh}}^{-1}{\left(\frac{Z}{A}\right)}^{1/n}$$

where σ is the flow stress, β is the temperature-independent material’s parameter, *n* is a stress exponent, *A* is a constant, and *Z* is the Zener-Hollomon parameter, defined by:

(2) $$Z=\dot{\bar{\varepsilon }}e^{Q/RT}$$

where $\dot{\bar{\varepsilon }}$ is the effective strain rate, *Q* is the activation energy, *T* is the absolute temperature (K), and *R* is the universal gas constant. For the hot extrusion of the AA6063 used in the present simulations, parameter values in Equations (1) and (2) were as follows [14,15]:

$$\text{β}=4\times 10^{-8\;}{\text{m}}^{2}/\text{N},R=8.314\text{ J}/\left(\text{mol}\cdot \text{K}\right),\;A=5.91\times 10^{9}{\text{ s}}^{-1},n=5.385,Q=1.416\times 10^{5\;}\text{J}/\text{mol}$$

**Figure 2:**
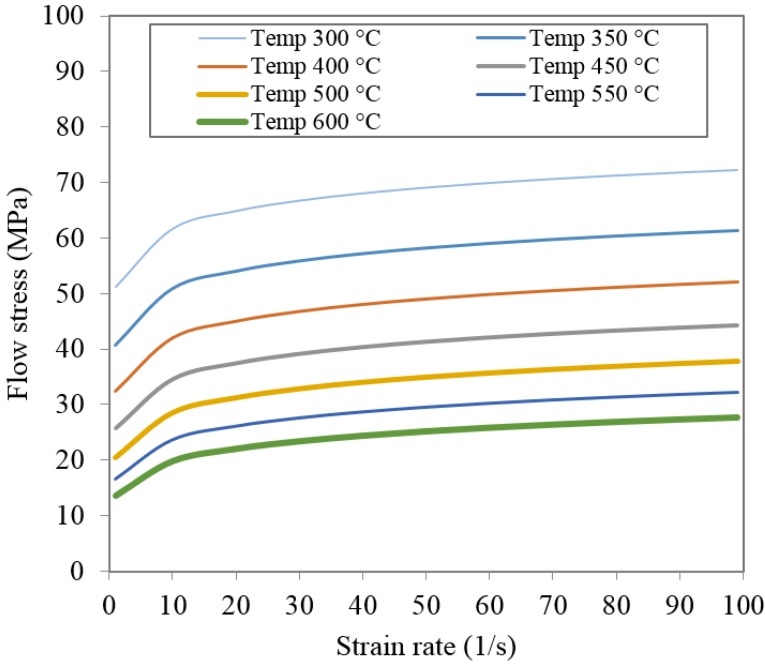
Flow stress of AA6063.

Figure 2 shows the flow stress-strain curves of AA6063. From the figure, the flow stress increases moderately with the increase of strain rate for a specific temperature. In contrast to this, the flow stress decreases strongly with the increase of temperature.

### 2.3. Finite Element Modeling Approach

The FEM simulations were performed with using DEFORM™ 2D. The temperatures of initial billet and tools were taken as 470 °C and 440 °C, respectively. The billet had a diameter of 200 mm. The die, container, and ram were modeled as rigid bodies. A friction coefficient of 0.85 was selected to exist between the billet and the container while it was assumed to be 0.4 between the workpiece and the die because of the sliding contact at the die’s bearing [16]. The ram speeds of 2, 4, 6, 10 and 15 mm/s were assigned as in the experiments.

The real extrusion condition of the hollow profiles is a case of 3D simulation. However, the welding line which is formed during the extrusion of the profiles can be modeled as a plane-strain. On the other hand, in the study of Xu [17], the 3D fixed pressure plate was defined in the same way as in 2D. However, the results at extrusion welding interface weren’t reasonable. According to Xu [17], the large scale differences between the elements in workpiece and plate is the main reason why the 3D simulation causes unreasonable results. As a result, a 2D plane-strain model instead of a complete 3D model was used in this study for suppressing possible alterations.

In the actual extrusion process, the pressure and effective stress at a given point change with different ram positions. Therefore, all of the positions of the ram should be considered. In this study, all of the step numbers during the ram’s travel were taken into consideration and, averages of the state variables were taken for approximately 500 step numbers. Further, the Plata and Piwnik’s criterion [18] was used to analyze the ratio of normal pressure to effective stress *(P/*σ*)* which is responsible for the strength of the welding in porthole extrusion:

(3) $$\int_{0}^{t}\frac{P}{\text{σ}}\text{d}t\geq COST$$

where *P* is the normal pressure on the welding line, σ is the effective stress, *t* is the contact time, and *COST* is a experimentally-determined constant value that assures sound extrusion.

## 3. Results and Discussion

**Figure 3:**
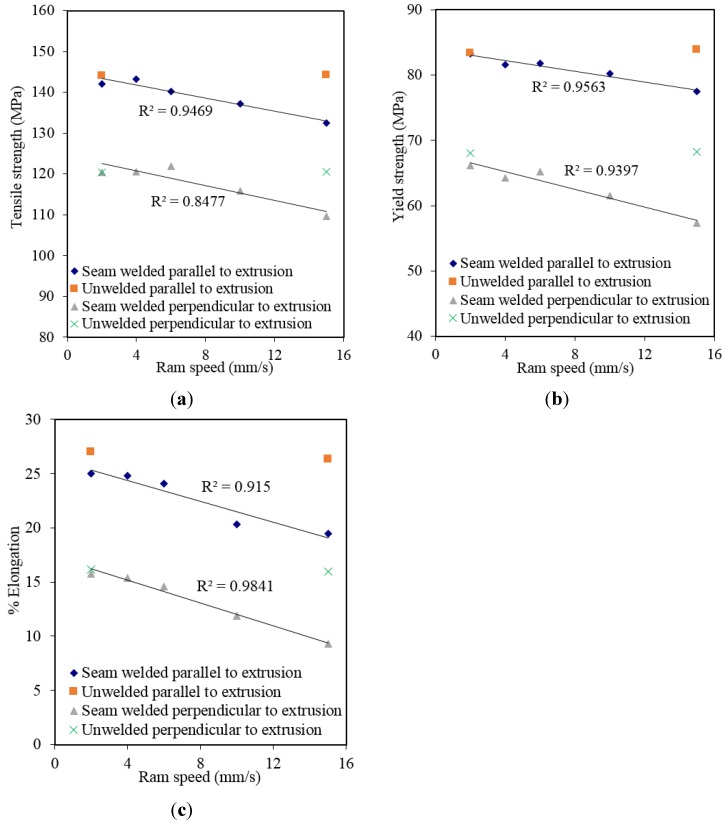
Effect of the ram speed on the specimens: (**a**) tensile strength; (**b**) yield strength; (**c**) % elongation.

Figure 3 graphically shows the results of effect of ram speed on ultimate tensile strength, yield strength and % elongation for the specimens prepared based on parallel to extrusion direction and perpendicular to extrusion direction. It can be seen from Figure 3 that increasing ram speed negatively influences the welding quality. However, the strengths of specimens parallel to extrusion direction were higher than the strengths of specimens prepared perpendicular to extrusion direction because the first deformed region in the tensile specimen under tension load is the middle of the specimen where seam weld located.

**Figure 4:**
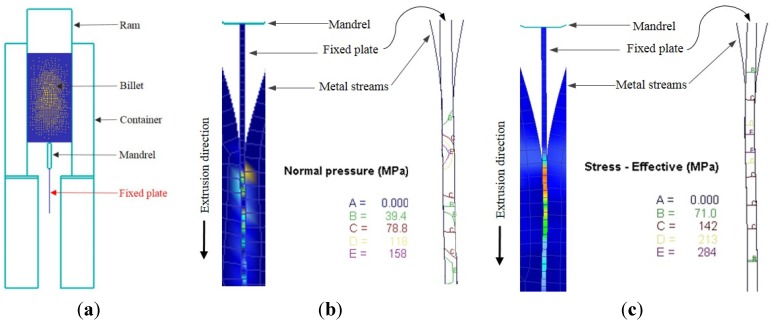
(**a**) The used fixed plate model [12,13]; (**b**) Normal pressure distributions on the fixed plate; (**c**) Effective stress distributions on the fixed plate (at a ram speed of 10 mm/s, a billet temperature of 470 °C, and for a constant simulation step number of 450).

**Figure 5:**
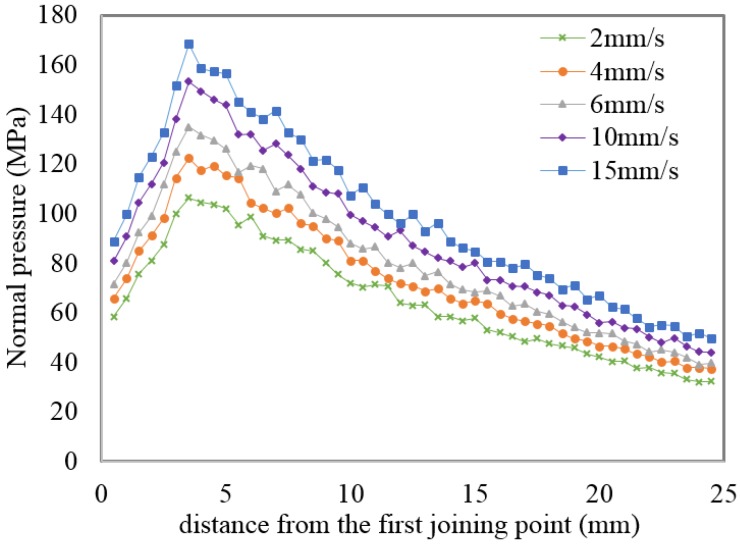
Normal pressure within the welding chamber at different ram speeds and at a billet temperature of 470 °C.

**Figure 6:**
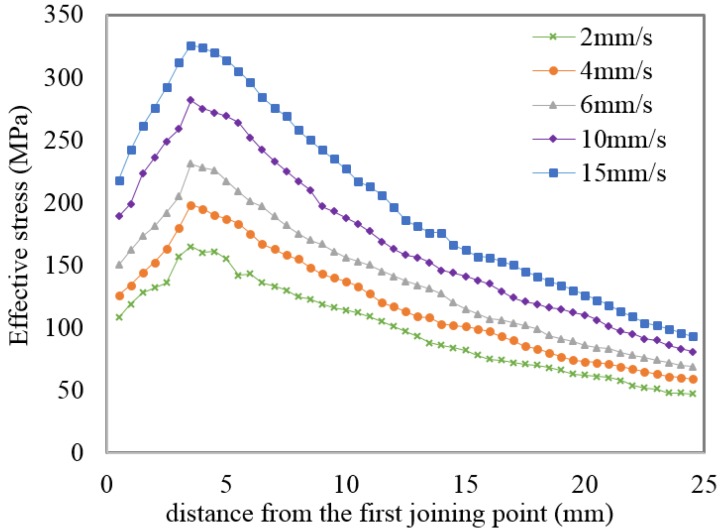
Effective stress within the welding chamber at different ram speeds and at a billet temperature of 470 °C.

Figure 4 shows FEM simulations of the normal pressure and effective stress distributions, respectively, on the pressure plate between the metal streams. In order to avoid the inclusion of too many figures to this paper, only simulations for a constant ram speed and billet temperature are shown in the figure. The main idea of the simulation with fixed plate model that was used is to display that the data for state variables can be seen on the fixed plate. As previously mentioned, FE packages cannot generate the local state variables at the extrusion welding interfaces. It can be seen from the Figure 4 that the normal pressure distribution inside the welding chamber and in the die’s bearing zone was not uniform during the extrusion process. This state in Figure 4 can also be seen from Figure 5 and Figure 6 which graphically shows the distribution of pressure and effective stress, respectively. It can be observed from the normal pressure and effective stress distribution that they reached their maximum levels at the almost same time inside the welding chamber while they have low values at the beginning of the welding chamber because the metal streams have just started to join and fill the welding chamber. This can be attributed to the complexity of the state of stress in the initiation of solid bonding, which requires a higher pressure to join the two metal streams.

**Figure 7:**
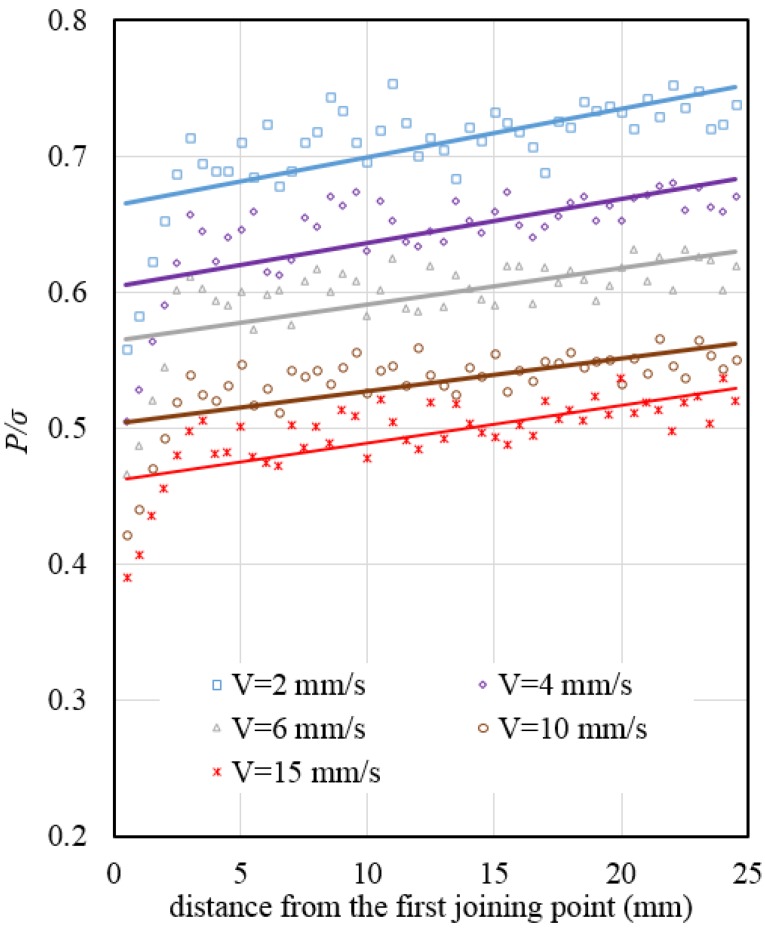
Ratio of normal pressure to effective stress (*P*/σ) at different ram speeds at a billet temperature of 470 °C.

Figure 7 was obtained by the ratio of normal pressure to effective stress *(P*/σ*) versus* the distance from the first joining point to the exit of the die based on the data from Figure 5 and Figure 6. As seen from Figure 7, the *(P*/σ*)* ratio for a constant ram speed remains almost constant for the entire length of the effective welding chamber and die’s bearing, except at the initiation of bonding. The initiation of bonding is weaker than the next bonding because the metal streams have just been joined.

**Figure 8:**
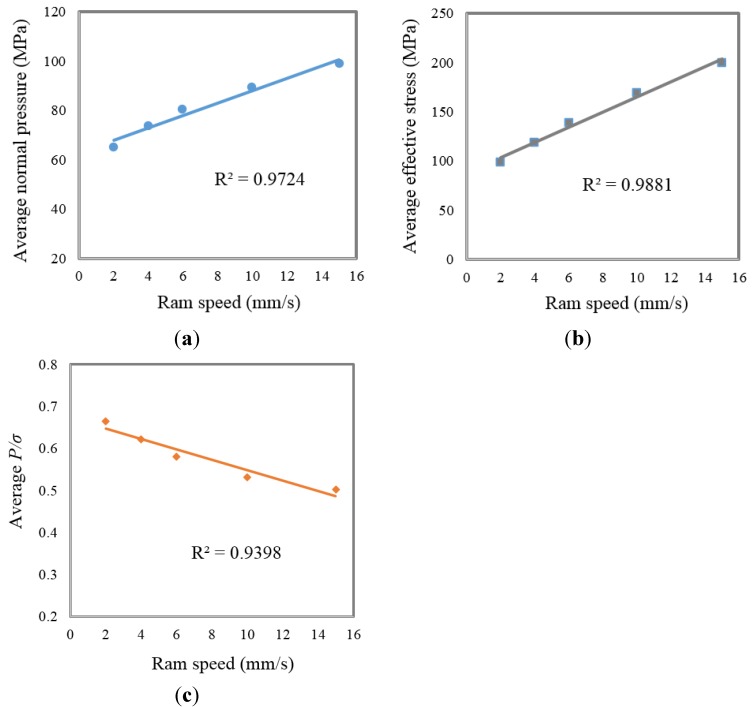
(**a**) Average normal pressure; (**b**) Average effective stress; (**c**) Average *P*/σ *versus* ram speed.

To clarify the effect of ram speed on welding quality, Figure 8a and Figure 8b (obtained from Figure 5 and Figure 6) and Figure 8c (obtained from Figure 8a,b), which show the average normal pressure, average effective stress and average *(P*/σ*)*, respectively, were arranged. It is clearly seen from Figure 8a,b that both the average normal pressure and effective stress increased as the ram speed increased. In contrast, the ratio *(P*/σ*)* which controls the quality of welding in Plata and Piwnik’s criterion decreased as the ram speed increased as seen in Figure 8c. In other words, the quality of welding was influenced negatively by the increasing ram speed. This is in a good agreement with the experimental results, which are graphically seen in Figure 3 which weaker extrusion welds occurred when the ram speed was increased. This reason of this case is explained by the limited time provided for the materials to bond within the welding chamber.

## 4. Conclusions

The effect of ram speed on extrusion welding was investigated experimentally and analyzed numerically. The main conclusions are as follows:

- Increasing ram speed negatively influences the seam-welded quality of AA6063. However, the strengths of the welded specimens parallel to extrusion direction are higher than the strengths of the welded specimen prepared perpendicular to extrusion direction.
- With the increasing ram speed in numerical simulations, *P* and σ increased. However, the ratio *P/*σ, which is responsible for the quality of welding in extrusion, was decreased. This case was verified by performed experiments, which show that the strength of specimens decreased with increasing ram speed.
- The distributions of *P* and σ within the welding chamber are not uniform. They are low within the beginning of the welding chamber due to the fact that the metal streams are just starting to be joined. After reaching their maximum values, *P* and σ decrease toward the exit of the die. However, the *(P*/σ*)* ratio remains almost constant toward the exit of the die.
